# Homing of mRNA-Modified Endothelial Progenitor Cells to Inflamed Endothelium

**DOI:** 10.3390/pharmaceutics14061194

**Published:** 2022-06-02

**Authors:** Denis Canjuga, Heidrun Steinle, Jana Mayer, Ann-Kristin Uhde, Gerd Klein, Hans Peter Wendel, Christian Schlensak, Meltem Avci-Adali

**Affiliations:** 1 Department of Thoracic and Cardiovascular Surgery, University Hospital Tuebingen, Calwerstraße 7/1, 72076 Tuebingen, Germany; denis.canjuga@uni-tuebingen.de (D.C.); heidrun.steinle@uni-tuebingen.de (H.S.); jana.mayer@alice.de (J.M.); ann-kristin.uhde@student.uni-tuebingen.de (A.-K.U.); hans-peter.wendel@med.uni-tuebingen.de (H.P.W.); christian.schlensak@med.uni-tuebingen.de (C.S.); 2 Center for Medical Research, Department of Medicine II, University of Tuebingen, Waldhörnlestraße 22, 72072 Tuebingen, Germany; gerd.klein@uni-tuebingen.de

**Keywords:** synthetic mRNA, EPCs, homing, engineering, angiogenesis, endothelium

## Abstract

Endothelial progenitor cells (EPCs) are one of the most important stem cells for the neovascularization of tissues damaged by ischemic diseases such as myocardial infarction, ischemic stroke, or critical limb ischemia. However, their low homing efficiency in the treatment of ischemic tissues limits their potential clinical applications. The use of synthetic messenger RNA (mRNA) for cell engineering represents a novel and promising technology for the modulation of cell behavior and tissue regeneration. To improve the therapeutic potential of EPCs, in this study, murine EPCs were engineered with synthetic mRNAs encoding C-X-C chemokine receptor 4 (CXCR4) and P-selectin glycoprotein ligand 1 (PSGL-1) to increase the homing and migration efficiency of EPCs to inflamed endothelium. Flow cytometric measurements revealed that the transfection of EPCs with CXCR4 and PSGL-1 mRNA resulted in increased expressions of CXCR4 and PSGL-1 on the cell surface compared with the unmodified EPCs. The transfection of EPCs with mRNAs did not affect cell viability. CXCR4-mRNA-modified EPCs showed significantly higher migration potential than unmodified cells in a chemotactic migration assay. The binding strength of the EPCs to inflamed endothelium was determined with single-cell atomic force microscopy (AFM). This showed that the mRNA-modified EPCs required a three-fold higher detachment force to be released from the TNF-α-activated endothelium than unmodified EPCs. Furthermore, in a dynamic flow model, significantly increased binding of the mRNA-modified EPCs to inflamed endothelium was detected. This study showed that the engineering of EPCs with homing factors encoding synthetic mRNAs increases the homing and migration potentials of these stem cells to inflamed endothelium. Thus, this strategy represents a promising strategy to increase the therapeutic potential of EPCs for the treatment of ischemic tissues.

## 1. Introduction

Cardiovascular diseases and the resulting complications, such as myocardial infarction, are among the leading causes of death worldwide. Injury and inflammation of the endothelial cells lining blood vessels, as well as the occlusion of blood vessels due to atherosclerosis and thrombosis, can lead to severe organ dysfunction and irreversible damage. Prolonged ischemia of the myocardium results in the irreversible loss of cardiomyocytes and vasculature, which are essential for maintaining cardiac integrity and function [1].

Endothelial progenitor cells (EPCs) are predifferentiated adult stem cells with the ability to differentiate into endothelial cells [2]. EPCs are mobilized from the bone marrow to the peripheral blood after ischemia or vascular injury [3,4,5]. The recruitment of EPCs occurs through the release of EPC-mobilizing cytokines, such as vascular endothelial growth factor (VEGF) [6], stromal-cell-derived factor-1α (SDF-1α) [7,8,9], or granulocyte-colony-stimulating factor (G-CSF) [10], to regenerate injured ECs and induce revascularization at the site of injury [11].

In recent years, numerous studies have investigated the therapeutic effects of EPCs in cardiac repair [12,13,14]. The homing of EPCs to sites of vascular injury or neovascularization requires a dynamic and multistep process of mobilization, chemotaxis, tethering, adhesion, and migration into subendothelial tissue [15]. In this process, the release of chemokines from ischemic tissue and the interaction of EPC surface molecules with their ligands on activated endothelium both play an important role in EPC homing. SDF-1α is a potent chemoattractant for EPC recruitment along a gradient toward ischemic tissue [16]. Our recent study [17] demonstrated the successful migration of EPCs towards SDF-1-mRNA-engineered EPCs. The function of SDF-1α is induced by binding to C-X-C chemokine receptor 4 (CXCR4), which is expressed on EPCs [18,19]. In addition to chemotaxis, the expression of the adhesion molecules P-selectin and E-selectin on activated endothelium plays an important role in EPC adhesion and migration [20,21]. The ligand of these adhesion molecules is P-selectin glycoprotein ligand 1 (PSGL-1), which mediates the attachment of circulating EPCs to inflamed endothelium [22].

However, previous clinical studies have shown that the low homing rate and low retention of circulating EPCs significantly limit the use of EPCs to promote angiogenesis. Thus, novel strategies are required to enable efficient treatment with EPCs. Therefore, various methods can be used to enhance and improve the homing of EPCs to desired areas, such as pretreatment of the target tissue or EPCs [23].

In recent years, the use of synthetic messenger RNA (mRNA) has attracted considerable interest due to several advantages [24]. Synthetic mRNAs are not integrated into the genome, avoiding the risks associated with insertional mutagenesis [25]. Furthermore, synthetic mRNAs are only transiently present in the cells until they are naturally degraded [26]. Thus, no footprints are left, and complications associated with permanent protein overexpression are prevented [27]. Compared to plasmid transfections, synthetic mRNA does not need to be delivered into the nucleus [28]. After the release of mRNA into the cytosol, the mRNA is immediately translated into proteins by cellular translation machinery under physiological conditions.

In this study, we investigate whether EPCs can be engineered using synthetic, modified mRNAs encoding CXCR4 and PSGL-1 to increase the expression of homing ligands on EPCs, and we analyze the homing potential of mRNA-modified EPCs to inflamed endothelium.

## 2. Materials and Methods

### 2.1. In Vitro Synthesis of Modified mRNA

pcDNA 3.3 vector (Aldevron, Fargo, ND, USA) that contained the coding sequence of murine PSGL-1 or CXCR4 was used as a template for the synthesis of DNA. The synthesis of DNA and the in vitro transcription (IVT) of mRNA were performed as previously described [17,29]. Briefly, to amplify the DNA sequence, a Hotstar HiFidelity Polymerase Kit (Qiagen, Hilden, Germany) was used according to the manufacturer’s instructions. For the PCR, 100 ng of plasmid DNA, 0.7 µM of the forward primer (5′-TTG GAC CCT CGT ACA GAA GCT AAT ACG-3′), and 0.7 µM of the reverse primer (T_120_-CTT CCT ACT CAG GCT TTA TTC AAA GAC CA-3′) (Ella Biotech, Martinsried, Germany) were used. The PCR products were generated using the following cycling protocol: an initial activation step at 95 °C for 5 min, followed by 25 cycles of denaturation at 95 °C for 45 s, annealing at 58 °C for 1 min, extension at 72 °C for 1 min, and a final extension at 72 °C for 5 min. After the DNA amplification, the PCR products were purified using a QIAquick PCR purification kit (Qiagen, Hilden, Germany) and were eluted in nuclease-free water (Qiagen, Hilden, Germany). The size and purity of the DNA were analyzed using 1% agarose gel electrophoresis at 100 V for 45 min and staining with 1× GelRed (Biotium, Fremont, CA, USA) in Tris-borate-EDTA (TBE) buffer.

Next, the IVT of the DNA into mRNA was performed using a MEGAscript T7 Kit (Life Technologies, Darmstadt, Germany) according to the manufacturer’s instructions. Therefore, 40 µL of the IVT reaction mix containing 7.5 mM adenosine triphosphate (ATP), 1.875 mM guanosine triphosphate (GTP), 7.5 mM 5-methylcytidine-5′-triphosphate (m5CTP) (TriLink BioTechnologies, San Diego, CA, USA), 7.5 mM pseudouridine-5′-triphosphate (ψ-UTP) (TriLink BioTechnologies, San Diego, CA, USA), 2.5 mM 3′-0-Me-m7G(5′)ppp(5′)G RNA Cap Structure Analog (ARCA) (New England Biolabs, Frankfurt am Main, Germany), and 40 U RiboLock RNase inhibitor (Thermo Fisher Scientific, Waltham, MA, USA) was prepared in 1× reaction buffer and 1× T7 RNA polymerase enzyme mix. The IVT reaction mix was incubated for 4 h at 37 °C. Afterwards, 1 µL TURBO DNase (T7 MEGAscript kit) was added to the IVT mix and incubated for 15 min at 37 °C to remove the remaining template DNA. Then, the mRNA was purified using a RNeasy Mini Kit (Qiagen, Hilden, Germany) according to the manufacturer’s instructions. The mRNA was dephosphorylated using 5 U/mL Antarctic phosphatase (New England Biolabs, Frankfurt am Main, Germany) at 37 °C for 30 min. The mRNA was purified again using a RNeasy Mini Kit, and the concentration was measured with a ScanDrop spectrophotometer (Analytic Jena, Jena, Germany) and adjusted to 100 ng/µL with nuclease-free water. The size and purity of the synthesized mRNA were analyzed using 1% agarose gel electrophoresis, as described for the DNA analysis. The mRNA was stored at −80 °C until use for transfection.

### 2.2. Cultivation of EPCs

Murine embryonic EPCs (T17b) [30] were cultivated in DMEM with high glucose and L-glutamine, containing 20% fetal bovine serum (FBS), 1× minimum essential medium (MEM) non-essential amino acid (NEAA) solution, 100 µM 2-mercaptoethanol, and 1% penicillin and streptomycin. All the reagents were obtained from Thermo Fisher Scientific (Waltham, MA, USA). The cells were cultivated at 37 °C with 5% CO_2_, and the medium was changed every 2 to 3 days. After reaching a confluency of 70–80%, the cells were detached using 0.04% trypsin, 0.03% EDTA, and a trypsin-neutralizing solution (TNS, 0.05% trypsin inhibitor in 0.1% BSA; PromoCell, Heidelberg, Germany). After centrifugation for 5 min at 300× *g*, the cells were seeded onto 0.1% gelatin (Sigma Aldrich, Steinheim, Germany)-coated tissue flasks or cell culture plates.

### 2.3. Cultivation of Human Umbilical Vein Endothelial Cells (HUVECs)

HUVECs (ATCC^®^, Manassas, VA, USA) were seeded in T75 cell culture flasks coated with 0.1% gelatin and were cultivated at 37 °C with 5% CO_2_ in Vasculife^®^ EnGS EC culture medium (CellSystems, Troisdorf, Germany) containing a VascuLife EnGS LifeFactors Kit, 50 mg/mL gentamicin, and 0.05 mg/mL amphotericin B (Thermo Fisher Scientific, Waltham, MA, USA). The medium was changed every 3 days. After reaching 80% confluency, the cells were detached using trypsin, EDTA (0.04%, 0.03%, PromoCell, Heidelberg, Germany), and TNS (PromoCell, Heidelberg, Germany).

### 2.4. Transfection of EPCs with Synthetic, Modified mRNA

To perform the transfection experiments, 1 × 10^5^ EPCs were seeded per well in a 6-well plate coated with 0.1% gelatin and incubated overnight at 37 °C. The next day, single mRNA transfections were performed with 1 µg PSGL-1 or 1 µg CXCR4 mRNA and 2 µL Lipofectamine^®^ 2000 (Thermo Fisher Scientific, Waltham, MA, USA) in 0.5 mL Opti-MEM I serum-reduced medium (Thermo Fisher Scientific, Waltham, MA, USA). Transfections with both the mRNAs were performed using 1 µg PSGL-1 and 1 µg CXCR4 mRNA with 4 µL Lipofectamine^®^ 2000 (mRNA cocktail). To generate mRNA lipoplexes, the transfection mix was incubated for 20 min at room temperature (RT) and then added dropwise to the cells. After 4 h of incubation, the transfection medium was replaced with 2 mL fresh cell culture medium per well and further incubated overnight at 37 °C. The cells incubated both with Opti-MEM alone and with transfection reagent (TR) served as controls.

### 2.5. Viability Assay

The impact of synthetic PSGL-1 and CXCR4 mRNA transfection on cell viability was investigated using a PrestoBlue™ assay (Invitrogen, Carlsbad, CA, USA). Therefore, 1 × 10^5^ EPCs were transfected with 1 µg PSGL-1 or CXCR4 single mRNAs or with the mRNA cocktail containing both mRNAs with 1 µg each for 4 h at 37 °C and incubated for 24 h in cell culture medium. Afterwards, the cells were washed once with Dulbecco’s phosphate-buffered saline (DPBS) without Ca^2+^/Mg^2+^, and 500 µL 1× PrestoBlue™ cell viability reagent was added per well. After 1 h of incubation at 37 °C, the fluorescence intensity of 100 µL supernatant was measured in triplicate at an excitation of 530 nm and an emission of 600 nm using a multimode microplate reader (Mithras LB 940, Berthold Technologies, Bad Wildbad, Germany).

### 2.6. Analysis of CXCR4 and PSGL-1 Expression by Flow Cytometry

The expressions of CXCR4 and PSGL-1 on the surfaces of the EPCs were analyzed 24 h after transfection with the synthetic mRNA. Therefore, 1 × 10^5^ transfected EPCs were detached, washed twice with 4% BSA/DPBS, and incubated with either PE rat anti-mouse CD162 (PSGL-1) antibody (BD Pharmingen, Heidelberg, Germany) or PE rat anti-mouse CXCR4 antibody (R&D Systems, Minneapolis, MN, USA) for 45 min at RT. After washing twice with 4% BSA/DPBS for 5 min at 500× *g*, flow cytometry analysis of 10,000 cells was performed using a FACScan system (BD Biosciences, Heidelberg, Germany).

### 2.7. Analysis of E-Selectin Expression on HUVECs

To analyze the activation of the HUVECs, 1 × 10^5^ HUVECs were seeded and cultivated at 37 °C with 5% CO_2_. After 24 h, the cells were activated with 10 ng/mL TNF-α (Sigma Aldrich, Darmstadt, Germany) for 4 h in culture medium. After 4 h, HUVECs with or without TNF-α treatment were stained with 5 µL mouse anti-human CD62E (E-selectin)-PE-conjugated antibody (Invitrogen, Waltham, MA, USA) according to the manufacturer’s instructions, and the E-selectin expression was analyzed using flow cytometry.

### 2.8. Chemotactic Migration Assay

The migration of modified EPCs towards a chemokine gradient was analyzed using a chemotactic migration assay. One day after the seeding of 1 × 10^5^ EPCs onto 0.1% gelatin-coated wells, transfection with 1 µg CXCR4 mRNA or mRNA cocktail (1 µg CXCR4 mRNA and 1 µg PSGL-1 mRNA) was performed for 4 h in Opti-MEM. Then, the transfection mix was removed, and cultivation medium was added for overnight incubation. The next day, 5 × 10^4^ mRNA-modified EPCs were seeded onto transwell insert membranes with 8 µm pores (Bio-One, Frickenhausen, Germany) coated with 0.1% gelatin. The transwell inserts were transferred to a 12-well plate containing 1% FBS serum-reduced culture medium and 50 ng/mL recombinant stromal-cell-derived factor 1 alpha (SDF-1α) (PeproTech, Hamburg, Germany) and incubated for 6 h at 37 °C. Subsequently, the transwell inserts were rinsed in DPBS with Ca^2+^/Mg^2+^ and fixed in ice-cold methanol (AnalaR NORMAPUR, VWR, Darmstadt, Germany) for 10 min. After washing with 0.5% BSA in DPBS, the cells at the bottom of the transwell inserts were stained with 1 µg/mL 4′,6-diamidino-2-phenylindole (DAPI, Sigma Aldrich) in DPBS for 10 min at RT. The detection of the migrated cells was performed using fluorescence microscopy (Axiovert135, Carl Zeiss, Oberkochen, Germany). The number of migrated cells was determined for 4 different regions of each insert using ImageJ 1.5.1 software.

### 2.9. Dynamic Adhesion Assay

The adhesion capacity of the mRNA-modified EPCs under flow conditions was analyzed after the mRNA transfection. Therefore, 1 × 10^5^ EPCs were seeded per well of a 6-well plate, transfected with the mRNA cocktail, and cultivated overnight at 37 °C and 5% CO_2_. The next day, the EPCs were stained with 2 µM PKH-26 (Sigma Aldrich, Darmstadt, Germany) in 1.5 mL DPBS without Ca^2+^/Mg^2+^ for 5 min at RT. The staining process was stopped by adding 1.5 mL FBS for 1 min, and the cells were washed twice in EPC medium. The cell number was adjusted to 1 × 10^5^ cells/mL and transferred to a 10 mL syringe.

A total of 1 × 10^5^ HUVECs were seeded onto 0.1% gelatin-coated 0.4 µ slides (Ibidi, Gräfelfing, Germany) and activated with 10 ng/mL TNF-α for 4 h at 37 °C with 5% CO_2_. The perfusion of the EPCs over the activated endothelium was performed at a flow rate of 0.11 mL/min with a shear force equivalent to 0.1 dyn/cm^2^. Images and video recordings were acquired after 1, 3, 5, and 7 min of perfusion using an Axiovert 135 microscope (Carl Zeiss, Jena, Germany) with an excitation of 550 nm. Analysises of the dynamic adhesion of mRNA-modified and unmodified EPCs to activated and nonactivated endothelium were performed using the MTrack function of ImageJ 1.5.1 software.

### 2.10. Single-Cell Atomic Force Microscopy (AFM)

AFM was used to measure the detachment force of the binding of mRNA-transfected and nontransfected EPCs to activated and nonactivated endothelium by measuring the force acting on the deflection of the cantilever and providing the signature of cell adhesion. A total of 5 × 10^4^ HUVECs were seeded per gelatin-coated plate (Ø 6 cm^2^), cultivated overnight, and activated with 10 ng/mL TNF-α for 4 h. The EPCs were transfected with a cocktail of CXCR4 and PSGL-1 mRNAs (1 µg each). After 24 h, the EPCs were detached, and a single cell was attached using a constant contact force of 1 nN for 30 s to an adhesive-coated tipless cantilever (All-in-One-cantilever D, 40 N/m nominal spring constant, Budget Sensors, Sofia, Bulgaria). The cantilever was coated with 10 µL Corning^®^ Cell-Tak in 300 µL of 0.1 µM bicarbonate buffer by incubating for 30 min at room temperature and rinsing with water.

The testing was performed in force spectroscopy mode by recording single force–distance curves at the position of interest (over a single HUVEC with neighboring cells) without laterally scanning the sample. The cantilever of the AFM (CellHesion 200, JPK Instruments, Berlin, Germany) was calibrated on the extend curve (vertical deflection), and its spring constant was determined using the thermal noise method of the data-processing software (JPK Instruments). The samples were measured at a maximum force of 800 nN with a pulling length of 90 µm and an extend speed of 5 μm/s in quadruplicate at four locations for each group. The deflection–detachment force curve was calculated using the data-processing software.

### 2.11. Statistical Analysis

The data are shown as mean + standard deviation (SD) or standard error of the mean (SEM). One- or two-way analysis of variance (ANOVA) was performed, followed by Bonferroni’s multiple comparisons test. To compare the means of two groups, a *t*-test was performed. All the analyses were performed using GraphPad Prism version 9.0.1. Differences of *p* < 0.05 were considered statistically significant.

## 3. Results

### 3.1. Analysis of CXCR4 and PSGL-1 Expression after the Transfection of EPCs with Synthetic, Modified mRNAs Encoding CXCR4 and PSGL-1

**Figure 1:**
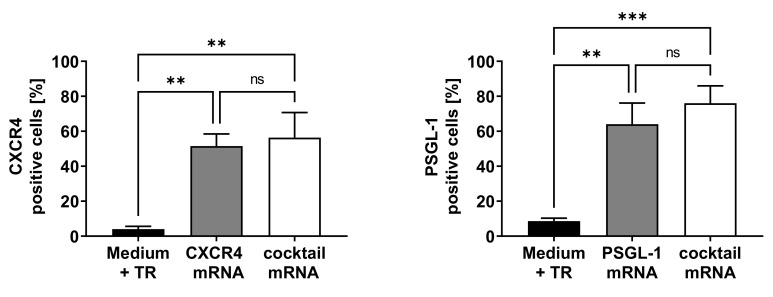
Analysis of CXCR4 and PSGL-1 expressions on the surfaces of EPCs after transfection with CXCR4 and PSGL-1 mRNA. A total of 1 × 10^5^ EPCs were seeded and transfected after 24 h with either 1 µg CXCR4, 1 µg PSGL-1 mRNA, or both mRNAs (mRNA cocktail). The CXCR4 and PSGL-1 expressions on the cell surfaces were analyzed 24 h post-transfection using flow cytometry. Cells treated only with medium and transfection reagent (TR) were used as negative control. Results are shown as mean + SD (*n* = 3). Statistical differences were determined using one-way ANOVA multiple comparisons, followed by Bonferroni’s multiple comparisons test (** *p* < 0.01 and *** *p* < 0.001; ns: nonsignificant).

After the successful synthesis of the synthetic mRNAs, the expressions of CXCR4 and PSGL-1 on the EPCs were determined. Therefore, single mRNA transfections with 1 µg CXCR4 or PSGL-1 or transfections with an mRNA cocktail consisting of 1 µg each of both the mRNAs were performed. The expressions of CXCR4 and PSGL-1 were measured using flow cytometry 24 h after transfection (Figure 1). After transfection with CXCR4 mRNA, 52% of the analyzed EPCs expressed CXCR4 on their cell surfaces, and transfection with PSGL-1 mRNA resulted in the expression of PSGL-1 on the surfaces of 64% of the EPCs. The cells transfected with the mRNA cocktail (CXCR4 and PSGL-1 mRNA) also showed elevated expressions of CXCR4 (56%) and PSGL-1 (76%).

### 3.2. Analysis of Cell Viability of EPCs after Transfection with Synthetic, Modified CXCR4 or PSGL-1 mRNAs

**Figure 2:**
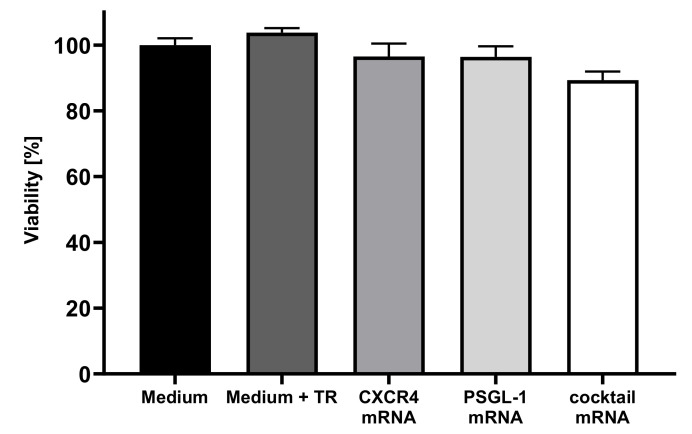
Analysis of cell viability of EPCs after transfection with CXCR4 and PSGL-1 mRNA. A total of 1 × 10^5^ cells were seeded and transfected after 24 h with 1 µg of CXCR4, PSGL-1 mRNA, or both mRNAs (mRNA cocktail). The viability was analyzed 24 h post-transfection using a PrestoBlue™ cell viability assay. The viability of cells incubated only with the medium was set to 100%. Results are shown as mean + SEM (*n* = 3).

To investigate the effect of mRNA transfection on the viability of EPCs, a PrestoBlue™ cell viability assay was performed 24 h after the mRNA transfection. The viability of cells incubated with medium was set to 100%, and the viability of the transfected cells was determined relative to the control. As shown in Figure 2, the transfection of EPCs with synthetic mRNA did not influence the cell viability.

### 3.3. Chemotactic Migration Assay of EPCs toward Chemoattractant SDF1-α

**Figure 3:**
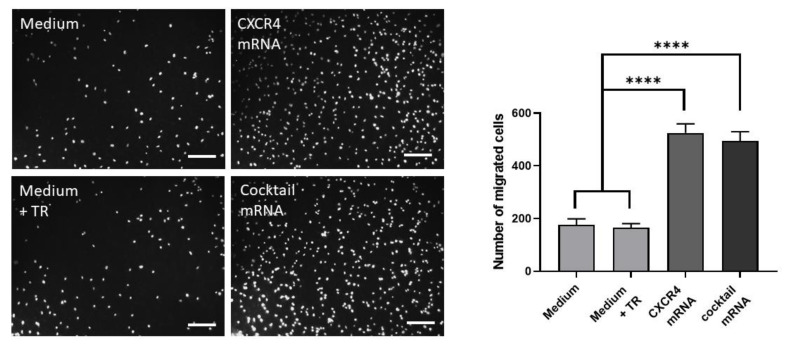
Chemotactic migration of mRNA-modified EPCs. A total of 1 × 10^5^ EPCs were transfected with either 1 µg CXCR4 mRNA or with an mRNA cocktail containing 1 µg CXCR4 and 1 µg PSGL-1 mRNA. After overnight cultivation, 5 × 10^4^ EPCs were seeded in 8 µm transwell inserts to analyze their migration capacity towards 50 ng/mL SDF-1α in a serum-reduced medium. Chemotactic migration was analyzed after 6 h at 37 °C by counting DAPI-stained migrated cells using ImageJ software. As controls, EPCs incubated with medium and medium containing transfection reagent (TR) were used. Scale bars represent 200 µm. Results are shown as mean + SD (*n* = 4). Statistical differences were determined using one-way ANOVA multiple comparisons, followed by Bonferroni´s multiple comparisons test (**** *p* < 0.0001).

An important chemoattractant for the homing of EPCs to a site of injury is SDF-1α, which directs EPC migration by binding to CXCR4. Therefore, the chemotactic migration potential of mRNA-modified EPCs was determined using a transwell migration assay. After transfection with 1 µg CXCR4 mRNA or the mRNA cocktail (1 µg CXCR4 and 1 µg PSGL-1 mRNA) and further overnight incubation, 5 × 10^4^ cells were seeded into transwell inserts and analyzed after 6 h of incubation. The migrated cells were stained with 1 µg/mL DAPI and automatically counted using Image J software (Figure 3).

EPCs transfected with CXCR4 mRNA (525 ± 10 cells) or the mRNA cocktail (CXCR4 and PSGL-1 mRNA; 495 ± 7 cells) resulted in a significantly increased migration rate compared with EPCs without mRNA transfection (medium: 168 ± 18 cells; medium + TR: 165 ± 12). This revealed enhanced and directed migration of the CXCR4-modified EPCs towards chemoattractant SDF1-α, indicating improved homing potential compared with cells expressing CXCR4 at basal levels.

### 3.4. Single-Cell AFM Analysis of the Adhesion of mRNA-Modified EPCs to TNF-α-Activated Endothelium

**Figure 4:**
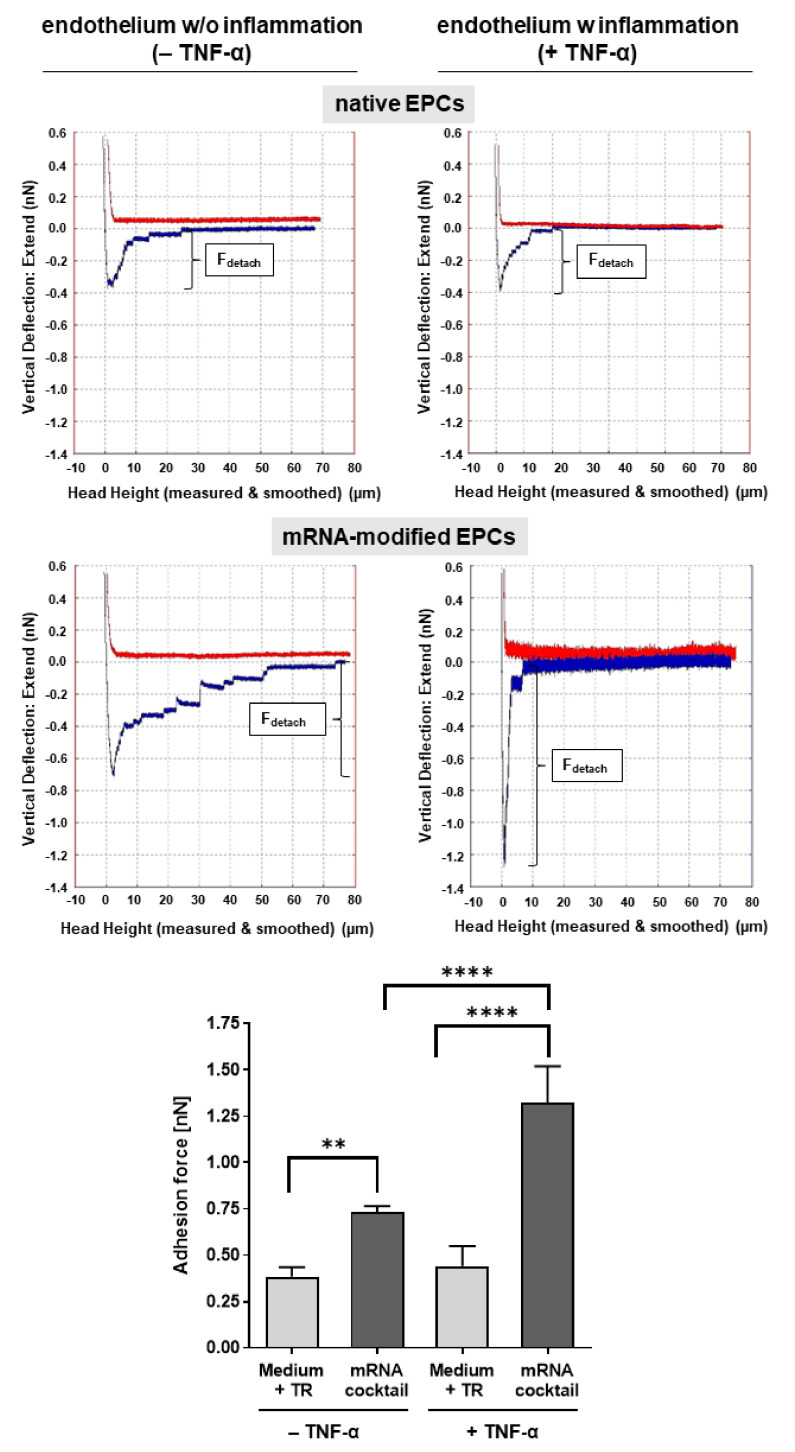
Analysis of the adhesion strength of EPCs to activated endothelium using single-cell atomic force microscopy (AFM). Representative histograms of the adhesion signature of CXCR4 and PSGL-1 mRNA (mRNA cocktail) modified and native EPCs to TNF-α-activated and nonactivated endothelium and the quantification of adhesion forces are shown. Bonds formed between the cell surface ligands (PSGL-1) and molecules on HUVECs (E-selectin) broke slightly with increasing force until the cell completely detached from the HUVEC. The maximum downward force of the cantilever’s tip is referred to as detachment force (F_detach_). (*n* = 4). Statistical analysis was performed using two-way ANOVA, followed by Bonferroni´s multiple comparisons test (** *p* < 0.01 and **** *p* < 0.0001).

The adhesion of mRNA-transfected and nontransfected EPCs to TNF-α-activated and nonactivated HUVECs was further analyzed to determine the binding strength of EPCs to the endothelium using single-cell AFM. EPCs modified with the mRNA cocktail encoding for CXCR4 and PSGL-1 were able to bind three-fold stronger to activated endothelium than unmodified EPCs, as demonstrated by the higher detachment force required to release the cell–cell interaction of a single EPC from activated HUVECs (Figure 4). In addition, mRNA-modified EPCs showed 1.8-fold stronger binding to activated endothelium than nonactivated endothelium.

### 3.5. Adhesion of mRNA-Modified EPCs on TNF-α-Activated Endothelium in a Dynamic Flow Model

**Figure 5:**
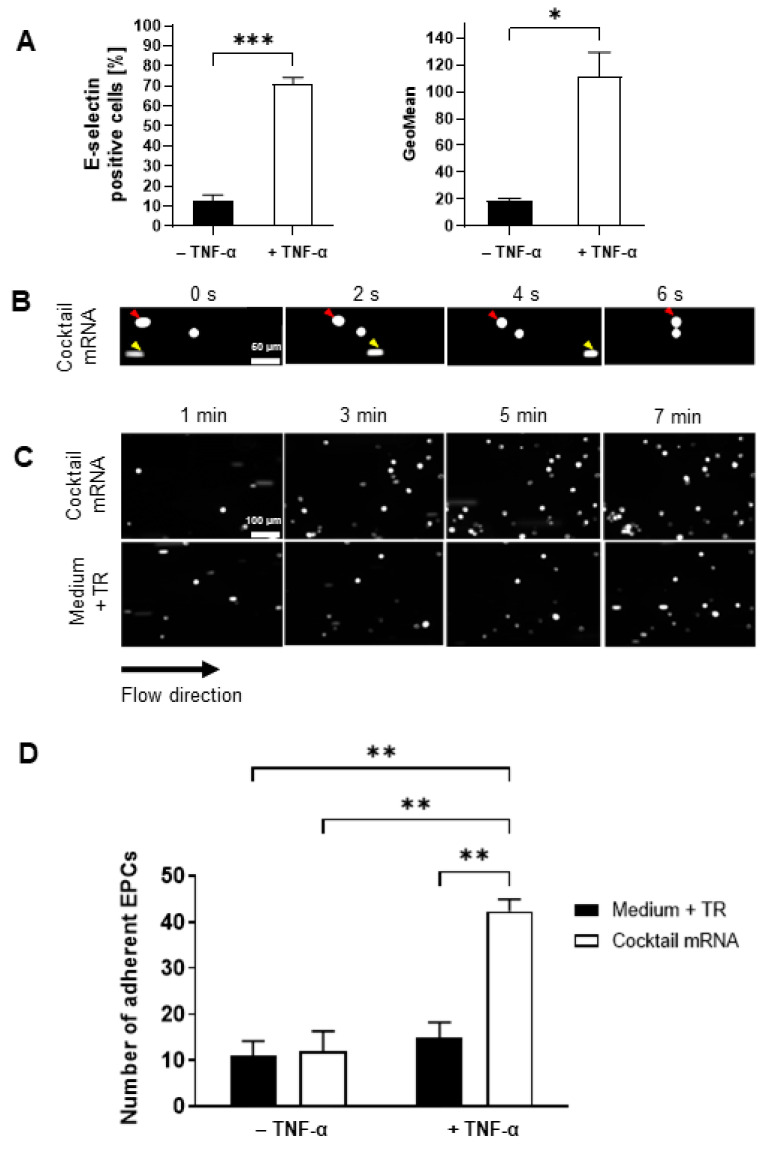
Dynamic adhesion of mRNA-modified EPCs to activated endothelium. (**A**) HUVECs were treated with 10 ng/mL TNF-α, and E-selectin expression was analyzed by flow cytometry to analyze the successful activation of the cells. (*n* = 3). Statistical analysis was performed using a *t*-test (* *p* < 0.05 and *** *p* < 0.001). (**B**) EPCs were transfected with an mRNA cocktail of CXCR4 and PSGL-1 mRNA (1 µg each). After 24 h, the cells were stained with PKH26 and perfused over TNF-α-activated HUVECs in a flow chamber at 0.11 mL/min and a shear stress of 0.1 dyn/m^2^. (**B**) Representative images of rolling EPCs are shown every 2 s. Red arrows indicate a slowly rolling cell, and yellow arrows point to a fast-moving cell not interacting with the activated HUVECs. (**C**) Representative images of recordings at 1, 3, 5, and 7 min after starting the flow. (**D**) The number of EPCs adhered to HUVECs after 7 min was quantified (*n* = 3). Statistical analysis was performed using two-way ANOVA, followed by Bonferroni´s multiple comparisons test (** *p* < 0.01).

The homing of mRNA-modified EPCs on inflamed endothelium was analyzed in a dynamic flow model. To stimulate inflamed conditions in vitro, the HUVECs were treated with 10 ng/mL TNF-α, which is a strong activator of the endothelium. As shown in Figure 5A, the treatment of HUVECs with TNF-α resulted in a significant upregulation of E-selectin expression compared with untreated cells, demonstrating the inflammatory response of the endothelial cells to TNF-α.

Next, mRNA-cocktail (CXCR4 and PSGL-1 mRNA, 1 µg each)-transfected and untransfected EPCs were perfused over the activated HUVECs at a constant flow rate of 0.11 mL/min with a defined shear force of 0.1 dyn/m^2^ to simulate the process of EPC adhesion. As highlighted in Figure 5B, the EPCs rolled on the activated endothelium and permanently adhered to it (red arrow). A fast-moving and non-adhering EPC in Figure 5B is indicated by the yellow arrows in the time course (0, 2, 4, and 6 s). Representative images of recordings (Supplementary Videos S1 and S2) at 1, 3, 5, and 7 min after starting the flow (Figure 5C) showed that mRNA-engineered EPCs interacted more strongly with the activated endothelium and attached to the inflamed endothelial cell surface in increased numbers, suggesting that adhesion molecules improved the rolling and adhesion of EPCs to the inflamed endothelium. In contrast, the unmodified EPCs adhered less to the activated endothelium. The quantification of adhered EPCs on the endothelium revealed that a significantly higher number of mRNA-transfected EPCs adhered to the activated endothelium within 7 min (cocktail mRNA: 42 ± 5 cells) compared with unmodified EPCs (Medium + TR: 15 ± 6 cells) (Figure 5D).

## 4. Discussion

The combined approach of cell therapy with synthetic, modified mRNAs represents a promising strategy to improve the revascularization and regeneration of ischemic tissues. In this study, we demonstrated the efficient modification of EPCs with synthetic mRNAs to express CXCR4 and PSGL-1 on cell surfaces to enhance their homing potentials to inflamed endothelium. The mRNA modification allowed EPCs to migrate and adhere more efficiently to the activated endothelium. Thus, this approach may represent a promising strategy for the treatment and regeneration of ischemic tissues.

EPCs play an essential role in postnatal vascular repair and remodeling through vasculogenesis and angiogenesis and offer a promising therapeutic option for vascular diseases [31]. Their mobilization from bone marrow and efficient homing to ischemic areas is, therefore, crucial for successful tissue regeneration and the limitation of tissue damage. Both the influence of tissue ischemia as an endogenous stimulus and the exogenous administration of cytokines (e.g., granulocyte macrophage-colony-stimulating factor, GM-CSF) were previously studied for the mobilization of EPCs [32]. The induction of ischemia in rabbit and mouse models resulted in an increased frequency of circulating EPCs [32]. In patients with acute myocardial infarction or coronary artery disease (CAD), increased numbers of EPCs have been mobilized and detected in circulation, as well as high VEGF levels, which have correlated with EPC levels [33,34].

The homing of EPCs is mainly mediated by various cytokines and chemokines. VEGF has been shown to promote mobilization and contribute to neovascularization [6,11,35]. Other growth factors or chemokines, such as angiopoietin-1 (ANG-1) or SDF-1α, have also been shown to stimulate EPC recruitment [35,36]. However, previous studies have shown that the functionality of EPCs derived from patients with CAD is impaired [37,38], indicating an insufficient ability to home to the ischemic regions even after mobilization from the bone marrow. To overcome these limitations and to improve angiogenesis, various methods, such as pretreatment of the target tissue or EPCs, have been investigated in the past using viral vectors encoding VEGF-A [39,40], HIF-1α [41], or plasmid-DNA-encoding SDF-1 [42]. SDF-1 is released from ischemic tissues and contributes to EPC homing by interacting with CXCR4 on the surface of EPCs [36]. Thus, in previous studies, induced mobilization of EPCs and enhanced angiogenesis have been observed after applications of SDF-1-encoding plasmid to the ischemic hind limbs of mice [43] or the ischemic myocardium of rats [44]. Although the use of cytokines is a promising approach to treat ischemia, side effects can occur due to the permanent expression of angiogenesis-inducing factors. Thus, a promising approach to circumvent the potential side effects of continuous exogenous protein expression is the use of transiently present, synthetic mRNA. In our recent study, synthetic mRNAs encoding the angiogenic factors ANG-1, SDF-1α, and VEGF-A were used to modify EPCs [17], and enhanced chemotactic migration and angiogenic potential were demonstrated in vitro and in vivo in a chicken chorioallantoic membrane (CAM) assay, particularly by using ANG-1-encoding, mRNA-modified EPCs.

In this study, the transfection of EPCs with synthetic mRNAs encoding CXCR4 or PSGL-1 resulted in an increased expression of the CXCR4 homing factor and the PSGL-1 adhesion molecule after single and combined (cocktail) applications of the mRNAs without detecting any negative effects on cell viability.

The chemokine SDF-1α binds to the CXCR4 chemokine receptor, which promotes homing towards damaged tissue [18,19]. Thus, significantly increased migration of the EPCs transfected with single or cocktail CXCR4 and PSGL-1 mRNA was observed compared with native EPCs, demonstrating the importance of CXCR4 in the homing process. In addition, single-cell AFM measurements showed that CXCR4- and PSGL-1-mRNA-modified EPCs adhered significantly more strongly to the inflamed endothelium than the unmodified EPCs, leading to higher forces required for the detachment of the EPCs from TNF-α-activated and nonactivated endothelium. The activation of the endothelium with TNF-α resulted in increased E-selectin expression, which led to a stronger binding of mRNA-modified EPCs to the activated endothelium than to the nonactivated endothelium.

A dynamic adhesion assay was used to simulate the homing and adherence of circulating EPCs to inflamed or injured vascular endothelium in vitro. EPCs transfected with the mRNA cocktail adhered and accumulated more on TNF-α-activated endothelium than on non-activated endothelium. Furthermore, no differences were observed on the nonactivated endothelium between mRNA-modified and unmodified EPCs, suggesting that modified EPCs efficiently homed only to the injured and inflamed endothelium. In contrast, on activated endothelium, EPCs modified with mRNA showed increased binding compared with unmodified EPCs.

In addition to chemokines guiding the cells to tissue injury sites, the adhesion molecules and their ligands also play a crucial role in the tethering of mobilized EPCs to the injured endothelium. EPCs express β_2_-integrins that can interact with the main ligand intercellular adhesion molecule 1 (ICAM-1) on the surface of the injured endothelium [45]. Thus, several studies have shown that increased ICAM-1 expression in ischemic muscle tissue leads to the increased recruitment of EPCs, angiogenesis, and the repair of damaged tissue [46,47]. These studies have indicated that stimulating the expression of molecules involved in the tethering of EPCs to inflamed endothelium can increase the binding and, thereby, improve the homing of EPCs at required sites. The transfection of EPCs with CXCR4 and PSGL-1 mRNA increased the adhesion forces and homing to activated endothelium in the dynamic adhesion assay, confirming the potency of this strategy. The enhanced binding of EPCs to E-selectin and P-selectin as a result of increased PSGL-1 expression was also demonstrated by Foubert et al. after the activation of EphB4 with an ephrin-B2-Fc chimeric protein [22].

Previous studies have shown that the number, function, and survival of EPCs are impaired in the elderly [48,49]. Thus, due to the impaired functionality and engraftment of EPCs, the clinical application of EPCs for cardiovascular cell repair therapies faces some obstacles in elderly patients [50]. To improve the function of EPCs and their repair capacity, they can be treated with growth factors, such as hepatocyte growth factor (HGF) [51], insulin-like growth factor (IGF-1) [52], or bone morphogenetic protein 4 (BMP4) [53]. Moreover, Li and colleagues showed that the transfection of EPCs with miR-326-5p significantly enhanced the angiogenic capacity of EPCs [54]. The transplantation of miR-326-5p-overexpressing EPCs improved cardiac function in an acute myocardial infarction model in mice. In a recent study, the treatment of EPCs with exosomes derived from SIRT1-overexpressing adipose-derived stem cells (ADSCs) [55] led to an increased expression of C-X-C motif chemokine 12 (CXCL12) and nuclear factor E2-related factor 2 (Nrf2) in EPCs from acute myocardial infarction patients and, thereby, improved the function of these EPCs. In another study, Masuda et al. demonstrated that the use of a new culture medium called the Quality and Quantity culture medium (QQ culture medium) for peripheral blood mononuclear cells (PBMNCs) could improve the vasculogenesis and angiogenesis functions of MNCs from healthy subjects [56]. Subsequently, this medium was also shown to increase the vasculogenic potential of diabetic human peripheral blood CD34+ cells compared with untreated cells [57]. In a recent study, Chruewkamlow et al. used this culture medium to analyze the influence on the functionality of PBMNCs derived from patients with chronic limb-threatening ischemia (CLTI) [58]. Culturing MNCs in QQ culture medium increased the percentage of CD34+/CD133+ cells (EPCs) and the anti-inflammatory cell population and resulted in improved angiogenesis compared to MNCs cultured in standard culture medium. Thus, the QQ culture medium could be used in combination with an mRNA-engineering strategy to increase the number of EPCs in the culture and compensate for the functional deficits of EPCs in clinical use. Moreover, the mRNA engineering of EPCs prior to transplantation could significantly improve the efficacy of EPC-based therapies by enhancing their homing to desired sites.

However, the synthetic-mRNA-based engineering strategy of EPCs used in this study requires the isolation and cultivation of EPCs from patients. This can be time-consuming and can hinder urgent applications. In addition, the quantities of EPCs in peripheral blood are low; thus, patients could be treated with cytokines such as G-CSF to increase the number of EPCs in the peripheral blood. However, the CD34+/CD133+ positive cells (EPCs) could also be isolated from bone marrow, adipose tissue, or peripheral blood using magnetic beads or flow cytometry to obtain higher numbers of EPCs that could be directly transfected with synthetic mRNAs and applied.

The use of synthetic mRNAs encoding adhesion molecules and their ligands, such as CXCR4 and PSGL-1, represents a novel method to increase the homing and adhesion of EPCs in ischemic tissue to overcome low retention rates after transplantation. Since exogenous protein expression is induced only transiently without the risk of genomic integration, potential side effects can be reduced, allowing the effective treatment of patients with ischemic cardiovascular diseases.

## 5. Conclusions

In this study, we demonstrated that the modification of EPCs with synthetic mRNAs encoding for CXCR4 and PSGL-1 improved the homing and adhesion of EPCs to inflamed endothelium. The increased expressions of CXCR4 and PSGL-1 were demonstrated after the single or combined mRNA transfection of EPCs with CXCR4- and PSGL-1-encoding mRNA. Furthermore, the improved migration and adhesion capacity of mRNA-modified EPCs was shown. Synthetic-mRNA-based cell engineering technology is a promising approach for the expression of desired receptors on stem cells to improve their homing potential to desired sites in the body and can enable the personalized treatment of various ischemic diseases such as myocardial infarction, strokes, or limb ischemia to improve the vascularization and regeneration of tissues.

## Supplementary Materials

The following supporting information can be downloaded at: https://www.mdpi.com/article/10.3390/pharmaceutics14061194/s1, Video S1: Native EPCs; Video S2: mRNA modified EPCs.
